# Nurse-led consultations reinforced with eHealth technology: a qualitative study of the experiences of patients with gynecological cancer

**DOI:** 10.1186/s12912-022-01104-9

**Published:** 2022-11-25

**Authors:** Mette Skorstad, Ingvild Vistad, Liv Fegran, Sveinung Berntsen, Berit Johannessen

**Affiliations:** 1 Department of Gynecology and Obstetrics, Sorlandet Hospital HF, Egsveien 100, 4615 Kristiansand, Norway; 2 Clinical Institute II, Medical Department, University of Bergen, Haukelandsveien 28, 5009 Bergen, Norway; 3 Department of Health and Nursing Science, University of Agder, Universitetsveien 25, 4630 Kristiansand, Norway; 4 Department of Sport Science and Physical Education, University of Agder, Universitetsveien 25, 4630 Kristiansand, Norway

**Keywords:** Nurse-led consultations, Follow-up care, Survivors of gynecological cancer, eHealth technology, Self-management, Empowerment

## Abstract

**Background:**

During the last decade, the health care profession has moved toward personalized care and has focused on the diversity of survivorship needs after initial cancer treatment. Health care providers encourage empowering patients to participate actively in their own health management and survivorship. Consequently, we developed and piloted a new follow-up model for patients at a Norwegian hospital, referred to as the Lifestyle and Empowerment Techniques in Survivorship of Gynecologic Oncology (LETSGO) model. Using LETSGO, a dedicated nurse replaces the physician in every second follow-up consultation, providing patients who have undergone cancer treatment with self-management techniques that are reinforced with eHealth technology via a specially designed app. Encouraging behavioral change and evaluating the late effects of treatment and recurrence symptoms are central components of self-management techniques. In addition, the app encourages physical activity and positive lifestyle changes, helps identify recurrence-related symptoms, and provides reminders of activity goals. This study aims to investigate experiences with nurse-led consultations supported by eHealth technology among the patients who piloted the LETSGO intervention.

**Methods:**

Semi-structured qualitative interviews were conducted to analyze the participants’ experiences with the LETSGO intervention after six to seven months.

**Results:**

The participants in the LETSGO pilot felt safe and well cared for. They thought the nurse was less busy than the doctors appear to be, which made it easy for them to share any cancer-related challenges. Many participants reported increased empowerment and confidence in recognizing symptoms of cancer recurrence, and participants who used the app regularly were motivated to increase their physical activity levels. However, the participants also experienced some limitations and technical errors with the app.

**Conclusions:**

Generally, the participants positively received the nurse-led consultations and eHealth technology, but an intervention study is required for further evaluation. In addition, the reported technical app errors should be resolved and tested prior to eHealth application implementation. Regardless, this study may be useful in planning personalized survivorship care studies.

**Trial registration:**

ClinicalTrials.gov,NCT03453788. Registration March 5, 2018.

**Supplementary Information:**

The online version contains supplementary material available at 10.1186/s12912-022-01104-9.

## Background

Traditional cancer follow-up care has been criticized for focusing more on recurrence detection and less on the physical and psychological consequences of cancer and cancer treatments [1]. In addition, the population continues to age, and more patients live with and beyond cancer because of improved treatments. Therefore, the demand for already strained health care resources has increased [2, 3]. Thus, traditional follow-up care for cancer must change. Cancer services have increasingly focused on the benefits of patient empowerment and self-management in the cancer survivorship trajectory [4, 5]. Patient empowerment enables patients to manage life aspects that are important to their health and health care [6], while self-management allows patients to take responsibility for their own well-being and lifestyle behavior, including medical and emotional issues that may arise after an illness [7]. Patient education regarding survivorship issues facilitates targeted, personalized health care that aligns with survivors’ needs and encourages them to take responsibility for their own health [4, 8, 9].

Endometrial cancer, the most common gynecological cancer, is associated with obesity and weight-related comorbidities, such as diabetes and hypertension [10]. Lifestyle changes, including physical activity and healthy nutrition, can enhance the quality of life and reduce the risk of cancer recurrence among survivors of gynecological cancers [11–13]. Thus, the promotion of patient education and self-management that includes a focus on physical activity can increase patients’ knowledge of and involvement in their disease management and can educate them about the key signs of recurrence. Informed patients who actively participate in their own care receive higher quality treatment than traditional approaches [8, 14]. However, consistent guidelines and strategies for implementing self-management among survivors of gynecological cancers in a clinical setting have yet to be developed.

Nurse-led consultations conducted via telephone or in person can alleviate time-restrained physician resources. In addition, these consultations lead to high satisfaction among patients, improving their trust in the health care provided and encouraging them to engage in their treatment and care plans [15, 16]. Furthermore, studies of nurse-led consultations report improved or equal quality-of-life outcomes among survivors of gynecological cancer compared to traditional follow-up care [16–20]. In addition, nurse-led counseling can improve sexual functioning in survivors of gynecological cancers [20, 21].

For example, a randomized controlled trial of survivors of ovarian cancer compared nurse-led physical activity coaching to traditional care controls [22], where the intervention group experienced a significant reduction in cancer-related fatigue and depression compared to the control group. Another randomized controlled trial compared follow-up care via nurse-led telephone consultations to traditional physician-led, hospital-based consultations in patients with FIGO stage I endometrial cancer [23]. When compared to the physician-led group, participants in the nurse-led group experienced greater patient satisfaction and suffered no detriment to anxiety and quality-of-life measures or delayed recurrence detection. Hence, the researchers argued that nurse-led consultations can replace or complement physician-led consultations without increasing patient anxiety or reducing satisfaction with the provided service.

Technology is rapidly evolving in the industrialized world, and it offers nearly unlimited possibilities. Electronic technology used for health care (i.e., eHealth technology) has significant potential to improve quality and safety in health care [24]. The use of eHealth in active cancer care and survivorship is also increasing [25], because it provides a way to tailor information, feedback, and self-monitoring options and to ease communication with health care professionals [26]. Various eHealth modalities, such as mobile phone applications, have shown promising results in improving patient engagement in self-management [27, 28].

Our research group developed LETSGO, a new follow-up model for patients with gynecological cancer. The main objective of this model is to increase patient involvement and personalized care by utilizing nurse resources and consequently reduce the number of consultations with oncologic physicians [29]. The LETSGO model schedules alternating nurse-led and traditional physician-led follow-up consultations, as described in Table 1, and the eHealth technology reinforces nurse-led consultations with a specially designed smartphone application (the LETSGO app) and activity tracker, as described in Table 2. We piloted the LETSGO model over a six-month period in a medium-sized Norwegian hospital, where one specially trained nurse conducted all the nurse-led consultations. Within three weeks of inclusion in the LETSGO pilot, the participants were scheduled for their first nurse-led consultations. The nurse used motivational interviews and behavioral change techniques to empower patients to be more active in their post-cancer care. The participants were scheduled for traditional physician-led consultations three months after their inclusion in the pilot, and they were scheduled for another nurse-led consultation after six months, which continued the same approach from the initial nurse-led consultations (see Tables 1 and 2).

**Table 1:** LETSGO Pilot

	**Time after Inclusion**	**Content**
First Nurse-Led Consultation^a^	Three weeks	Information, assessment, and handling of late effects (physical and psychological)
		Development of coping strategies
		Suggestions for beneficial lifestyle changes
		Discussions regarding social network, work, or relationship concerns
		Introduction to LETSGO app and Garmin activity tracker^b^ (provided free of charge)
		Goal setting for physical activities
		Instructions on manually logging counted steps from the activity tracker into the LETSGO app
Physician-Led Consultation^c^	Three months	Assessment of symptoms
		Physical examination
		Vaginal ultrasound
		Discussions of survivorship concerns
		Reinforcement of behavioral changes initiated by nurse
Second Nurse-Led Consultation^a^	Six months	Assessment of symptoms and survivorship concerns
		Evaluation and adjustment of behavioral change goals

^a^Each consultation lasted 45 min

^b^Introduced at first nurse-led consultation and maintained until end of study

^c^Each consultation lasted 30 min. Additional treatment modalities/tests were applied upon indication

**Table 2:** LETSGO Application Content

Sections	Contents
**Contact Information**	Telephone number and the nurse’s hours of availability for contact
**Disease Information**	
Treatment	Information, illustrations, videos
Signs of Recurrence	
Late Effects	
**Lifestyle Advice**	
Physical Activities	Information, illustrations, videos
Relaxation Activities	
Smoking Cessation	Information
Nutrition	
**Activity**	
Exercises	Suggestions for exercise programs of various levels with illustrations and videos
Goal Setting	Participant-reported activity goals for selected activities (minutes)
Activity Graphs	Display of activities performed (days and weeks), manually logged by the participants
**Patient Forms**	
Daily Walking Steps	Steps counted by activity tracker, manually logged by the participants
Minutes Spent on Activities^a^	Registration of activities (minutes)
Symptom Scores^b^	Questionnaire with 10 recurrence-specific questions, each rated by participants as “Not at All,” “A Little,” “Quite a Bit,” and “Very Much” Alarm and instruction to call the study nurse (telephone number provided in the app) if the answers indicate recurrence based on a predefined algorithm and threshold

^a^The app provided a weekly reminder to register activity

^b^The app provided a monthly reminder to submit the questionnaire

There are promising results of nurse-led care and eHealth technology among cancer patients, but qualitative research on these approaches is limited among patients with gynecological cancer. As the LETSGO trial piloted a novel follow-up model for patients with gynecological cancer, the aim of this study was to investigate the experiences with nurse-led consultations supported by eHealth technology among the patients who piloted the LETSGO intervention.

## Methods

### Study design

For our qualitative study, we conducted semi-structured interviews with participants in the LETSGO pilot based on the Consolidated Criteria for Reporting Qualitative Research [30]. We developed a thematic interview guide that focused on the participants experiences with the nurse-led consultations and the mobile application (see Appendix 1).

Following Lindseth and Norberg’s (2004) method, two researchers interpreted the data using the phenomenological hermeneutic approach [31]. Lindseth and Norberg explained, “In this tradition it has become obvious that essential meaning is something with which humans are familiar in the practices of life, and this familiarity has to be expressed through the way of living, through actions, through narratives and through reflection” (31, p. 146). In research, the lived experience (the phenomenological approach) must be fixed in the text, and the narratives subsequently require interpretation (the hermeneutical approach) [31, 32].

### Participants

Treating specialists invited the participants in the LETSGO pilot to participate in the interview study. All participants received written information regarding the study and consent forms. The study used the following inclusion criteria: patients who were enrolled in the LETSGO pilot, who understood and read Norwegian, who had no cognitive barriers, and who provided informed consent. The interview study did not have any exclusion criteria. Of the 17 eligible participants, 12 patients accepted the invitation and participated in the interview study. Two of the patients who declined participation did not wish to be interviewed, and the other three patients did not provide a reason.

### Data collection

Two female researchers who did not know the patients conducted face-to-face interviews in a hospital setting between October and November 2018. The primary interviewer (BJ) was a nurse and associate professor from a nearby university, and the secondary interviewer (MS) was a medical doctor and Ph.D. candidate from the hospital in which the study was conducted. The secondary interviewer was present in all the interviews and asked supplementary questions for a better understanding when necessary. All interviews were audio-recorded and transcribed verbatim, and each interview lasted between 18 and 41 min (median = 25.5 min). The semi-structured interview guide covered topics related to the nurse-led consultations, the LETSGO mobile app, and the participants’ physical activities during the pilot period. The interviewers asked questions regarding these topics and instructed the participants to describe their experiences freely. The interviewers encouraged further narration and reflection by asking such questions as “What happened next?” and “What did you feel?” The participants steered the conversation without prompting from the interviewers [31, 33].

### Data analysis

We used the phenomenological hermeneutic approach described by Lindseth and Norberg for data analysis [31]. In the first phase, we performed a naïve reading of the transcribed interviews. To capture the full meaning, we read the text multiple times with an open mind to allow it to speak to us [31].

During the second phase, we used Lindseth and Norberg definition of a theme: “A theme is a thread of meaning that penetrates text parts, either all or just a few. In order to capture this meaning of lived experience we do not formulate the themes as abstract concepts, but rather as condensed descriptions. We formulate them in a way that discloses meaning” (31, p. 149). Thereafter, we performed structural analyses to identify the descriptions and formulate and organize these into themes.

During the third phase, we summarized and reflected on the themes in relation to the research questions and the study context [31]. We developed this comprehensive understanding by considering the authors’ preconceptions, naïve readings, structural analyses, and relevant literature. When any doubt or disagreement arose, we reviewed the transcribed interviews and engaged in new discussions, and we eventually reached a consensus for all interpretations. This repetitive process enabled us to develop a broader understanding of the patients’ experiences with the nurse-led consultations and the app, and the analytical process resulted in four themes.

### Ethical considerations

The study conformed to the principles of the Declaration of Helsinki. All participants received verbal and written information about the qualitative study methods and aims, and they consented to participate in writing. The participants were free to withdraw their consent at any time, without justification and without consequences to their follow-up care, and all were given the opportunity to choose to be interviewed by the primary interviewer only. The Regional Committee for Medical and Health Research Ethics of South East Norway (2017/2195) approved the study, and it was registered at Clinicaltrials.gov (NCT03453788) on March 5, 2018.

## Results

### Study population

The 12 interviewed participants were 35–74 years old and had been treated for uterine, ovarian, cervical, or vulvar cancer of FIGO stages I, II, or III. The median age was 51.5 years. Eleven patients had received surgical treatment, half had undergone additional chemotherapy, and some had received radiation therapy. Most women in the pilot had finished their treatment less than two years prior to their participation therein, and some participants had previously been treated for cancer recurrence, with no signs of active disease.

### Interview results

**Fig. 1:**
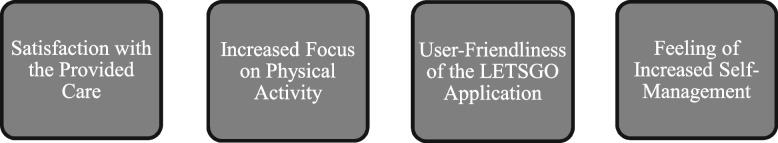
Interview themes

The participants explained that they participated in the study to support research and development in the area of cancer care. As we considered the results from the analyses, we divided them into four themes (see Fig. 1).

These themes illustrate that although the app experienced some technical issues, the patients gained useful knowledge of cancer survivorship and lifestyle changes while feeling reassured and well cared for in the LETSGO pilot. The theme “satisfaction with the provided care” highlights that the participants trusted the nurse’s expertise and care, while the theme “increased focus on physical activity” reflects the participants’ expressed motivation to become more physically active. Further, the theme “user-friendliness of the LETSGO application” identifies perceptions that the app was an easily accessible information tool, but also problems the participants encountered with the app software, and the theme “feeling of increased self-management” captures the participants’ perceptions of their ability to recognize concerning symptoms. The participants primarily discussed their experiences with nurse-led consultations.

### Satisfaction with the provided care

When asked about their overall opinions on partially nurse-led consultations and the use of eHealth technology, the participants expressed the importance of feeling safe. The participants appeared open-minded and to have great trust in the health care system. The nurse-led consultations were perceived as an appropriate and useful alternative to traditional physician-led consultations, and the participants found it easy to initiate contact with the nurse. They described the nurse as educated and qualified, and they felt they could discuss physical, psychological, and lifestyle concerns with her. The participants found it comfortable and soothing to see the same nurse when they returned for consultations, and the relationship they had established with the nurse was relaxed, as stated by a participant:

*“She had lots of knowledge, and I thought it was good to have someone to talk to. It is nice to know you are taken seriously.”* Participant 11

During the nurse-led consultations, the participants discussed achievable physical activities based on their interests and abilities. They were happy to learn that even small increases in activities may produce health benefits. Because of the nurse’s invitation to present concerns freely, the women could find the nurse-led consultations too short in duration to discuss their many survivor-related issues adequately. If having the perception that discussing concerns with a doctor might interfere with the doctor’s limited resources, the participants found it easier to talk with the nurse. Further, the option of calling a nurse between visits if concerns arose could be relieving, and the nurse was regarded as a screening gateway for symptoms that could spare physician resources if examinations were considered unnecessary:

*“When you know that if there is something and you can meet a nurse . . . then you don’t have to steal time from the doctor, if you don’t really need it.”* Participant 2

The fact that the app provided a contact number if the participants had questions or worries was appreciated, as it provided the opportunity to relieve anxiety related to participant concerns and gave a pleasing feeling of being taken seriously.

Reflections on the need for physical examinations varied. The participants expressed relief in not having to undergo more than one physical examination in a six-month period, and they felt confident in replacing some physician-led consultations with nurse-led consultations. However, more frequent physical examinations could also be perceived as reassuring:

*“I just think that all of a sudden there can be something there that you haven’t noticed. Like how they found [the malignant tumor] in my case, and I hadn’t noticed.”* Participant 8

While some participants believed they did not need nurse-led appointments between the physical examinations, others found it reassuring to talk to a nurse, and they felt confident that the nurse would consult the physician if necessary.

### Increased focus on physical activity

The encouragement to set a weekly activity goal and log achievements in the app motivated the women to become more physically active. However, the app’s physical activity module could be considered too basic and uninspiring compared to other training apps. For example, participants could enter only one weekly goal into the app, which limited the participants’ focus to that one activity. Furthermore, some of the younger participants wanted a graphic display of their achieved activities. These limitations could lead to discontinued use of the LETSGO app’s physical activity segment. In contrast, the participants appreciated the activity tracker, reporting that it motivated and encouraged them to walk or run, which helped them reach their daily step goals:

*“I used the activity tracker. So, if I hadn’t walked enough steps to meet the goal I’d set, then I just went for another walk to reach my goal.”* Participant 6

The participants reflected on how using the activity tracker not only increased their own physical activity but also motivated others around them to become more physically active. Those who refrained from using the activity tracker faulted the device’s design and its inability to register steps from bicycling.

### User-friendliness of the LETSGO application

Overall, the app was considered user-friendly and easily accessible. It provided concise information on the cancer disease, as well as links to recommended websites if the participants wanted further information:

*“I read everything. I think the information was excellent—good to know that there’s a place to obtain valid information, not just Google.”* Participant 4

Usage of the LETSGO app varied among participants. Regular use of the app was prominent, but consistently reading and hearing about the disease could also make participants feel overly conscious of and defined by the disease:

*“You don’t get healthy, reading about illnesses, you know.”* Participant 7.

There were different opinions regarding the app’s content. Many participants wanted the app to contain more detailed information on their disease, and they thought the sections regarding nutrition advice and relaxation exercises were not as useful.

During the interviews, the participants identified several software issues with the app. One specific issue limited positive encouragements from the app. In addition, physical activity had to be manually entered into the app before noon the following day. If the participants entered their achievements too late, they did not receive credit for reaching their goals. The participants also emphasized that it was inconvenient that they had to enter the number of steps from the activity tracker manually, and they shared ideas on useful app improvements:

*“Ideally, I wish the activity tracker was connected to the app. If I wanted to really evaluate activities and get motivated and such, then I used the Garmin app as well to see how far I had run.”* Participant 10.

It appeared that monthly reminders to submit answers to the recurrence-specific questions could fail to appear on the participants’ mobile device screens. There could be app freezes, or the participants could be dismissed from the app during the symptom evaluation. In these situations, the participants perceived the app as a less useful tool for helping them evaluate whether their current health status was concerning with regard to disease recurrence.

### Feeling of increased Self-management

Knowing what to look for after participating in the nurse-led consultations and engaging in monthly symptom ratings in the app relaxed the women and made them feel safe. The participants reported that they had gained new and important insights regarding their cancer diagnosis:

*“When I finished the cancer treatment, I didn’t know that symptoms were an important way of discovering a recurrence. I thought that it would be by a CT scan and examinations at the hospital, I mean, what the doctors do. But then I was taught to be more aware of my body.”* Participant 12

Similarly, the app was experienced as reassuring, and the regular symptom questions provided a feeling of security, mainly because symptom scoring gave the participants an opportunity to rate their own health condition:

*“Aha, that could be a sign of something wrong. I didn’t know that!”* Participant 9.

The participants also thought that knowing what to do and whom to call was reassuring. Participants who reported feeling healthy and cured tended to disregard information about their diagnosis and found the symptom questions irrelevant. The participants expressed that it was important for the nurse to provide a clear goal for the appointments, such as the objective of empowering participants in self-managing the physical and psychological aspects of their cancer survivorship. Those who considered themselves talkative were concerned that their talkativeness prevented the nurse from discussing scheduled topics during their consultations. Although the participants expressed overall confidence in the follow-up model, not everyone felt prepared to take responsibility for monitoring the potential symptoms of cancer recurrence:

*“You are supposed to monitor symptoms yourself, but how can you monitor your own genitals? You may be able to see bleeding or if you gain weight, but you can’t check yourself.”* Participant 8

A suggested improvement from a participant was for health care providers to receive and evaluate the symptom scores and physical activity achievements the participants submitted:

*“I would have liked if what I registered in the app was seen by someone on the other end. One believes that it will be sent out, and when it isn’t, then it’s not that valuable.”* Participant 3

## Discussion

The participants felt safe during the nurse-led follow-up consultations, and the communication with the nurse was perceived as valuable because the nurse seemed experienced and was not as busy as physicians appeared. This result aligns with a study conducted in the UK, which reported that survivors of ovarian cancer with three years of nurse-led telephonic follow-up care found that “time was never an issue” and that “somebody was looking out for [them]” [34]. Researchers who assessed survivors of endometrial and lymphoma cancer also found nurse-led appointments to be helpful, because the survivors’ experiences with nurses contrasted their perception of physicians as being too busy or less interested when contacted for support and reassurance [16, 23, 35]. Our participants found the nurse-led consultations and the app informative, and several of these services could correct the misconception that only physical exams or imaging scans reveal recurrences. The interviewed participants perceived the app’s monthly reminder to rate recurrence-associated symptoms in the app, as well as the advice to call the treating nurse if the entered symptoms related to an increased risk of recurrence, as useful and reassuring. The participants reported that learning to monitor their own bodies and health for symptoms could help them identify recurrences or other concerns that they should discuss with their health care providers. Our findings indicate that learning about cancer, expressing concerns, and deciding on survivorship care are important factors in increasing the empowerment of cancer survivors [36].

However, not all LETSGO participants understood the empowerment aspect of the app, and the app could be disregarded by participants who felt well and that they did not need it. These patients may have dismissed beneficial knowledge of alarming symptoms. If a recurrence was to occur later, the lack of knowledge for these participants could possibly delay their contact with health care providers. In addition, some patients may not want to know all the details of their illness, and some perceive regular reminders of their illness as counteractive to rehabilitation. Providing more information to patients who cope in this manner may increase their stress levels [37]. Consequently, information must be tailored to match the needs of survivors of gynecological cancer to prevent the infliction of harmful stress [38].

Because eHealth technology can identify a problem and guide appropriate action, it is a powerful and expanding technological tool that may be beneficial in health care settings [39]. This technology can reduce the number of face-to-face consultations, easing overburdened health care services in a cost-effective and useful manner [40]. Using eHealth technology has led to improved survival [41] and reduced hospital admissions [41, 42] among cancer survivors. However, there is limited knowledge of the benefits of eHealth technology for survivors of gynecological cancer. Generally, patients welcome eHealth technology to support empowerment and self-management [43]. Overall, the participants in the present study, regardless of their cancer diagnosis, found the app and activity tracker useful, although younger participants thought the app was limited and uninspiring compared to established nonmedical apps. This result highlights the potential value of additional studies on the use of eHealth technology among survivors of gynecological cancer.

The participants’ feedback regarding the failures of the app was also useful. Technical issues that cause the app to malfunction may fail to alert survivors of important actions or result in dissatisfaction with the app, leading to discontinued use [44, 45]. In addition, it is important to balance easy-to-use functionality that enables use by patients who are less accustomed to technology with visually appealing content that enhances user engagement [44]. Studies conducted among health care professionals have indicated an increased workload is a major concern with implementing eHealth technology in clinical practice [43]. Thus, it is important to test the app extensively, solve any problems, and establish well-functioning technical support before implementing the app in daily practice [25].

The pilot participants were instructed to enter symptoms and physical activity achievements into the LETSGO app. However, not all found this exercise meaningful, knowing that the data were not shared with the responsible health care providers. Similarly, the participants could fail to perceive patient education as an important aspect of logging physical activity achievements. Thus, to ensure patient commitment and the optimal effects of self-management interventions in a clinical setting, it is important to remind patients repeatedly why symptom evaluation and physical activity goal setting are encouraged. Another raised concern was that reading and hearing about one’s disease may make patients feel overly conscious of and defined by their disease, despite feeling well. This is an important aspect that must be considered when planning future follow-up care. This viewpoint is consistent with reports from other studies that have identified a significant variance among cancer survivors regarding their need for information [46, 47], and it indicates the importance of not inflicting greater worry or stress through patient education, even when well-intentioned. Nurse-led consultations that use motivational interviews to encourage patient reflection on lifestyle changes and survivorship needs, combined with eHealth technology, can be an effective strategy for personalizing care and optimizing survivorship trajectories. However, this model of follow-up care after cancer treatment should be tested in larger intervention studies.

### Strengths and limitations

A strength of the present study was the diversity in the participants’ demographical characteristics in terms of their age, gynecological diagnoses, cancer stage, and comfort level with smartphone use. The fact that two researchers interviewed the participants could be argued as either an advantage or a disadvantage. The participants could potentially feel uncomfortable if a power imbalance is perceived when meeting two interviewers, and the opportunity for rich data collection could be lost [48]. On the other hand, a second interviewer may facilitate a better exploration of the understanding and meaning behind the narratives [48]. The researchers who analyzed and discussed the data were from different academic backgrounds, and this researcher triangulation may have resulted in a broader understanding of the participants’ reflections.

One threat to the trustworthiness of the study is if the participants perceived that the interviewers, who represented the hospital that initiated the study in review, expected a favorable evaluation from them. In such a situation, the participants may sugarcoat their experiences and not fully describe their reality [49]. However, we mitigated this factor through several strategies. First, none of the interviewers knew the participants, and the participants were reminded of the confidentiality and anonymity procedures. Second, the interviews were performed outside earshot of others to help put the participants at ease [49]. Finally, the participants were encouraged to report any negative experiences to help assess whether the follow-up model was beneficial for future survivors. The pilot was conducted at a single practice site in Norway. Thus, transferability of this study to other populations may be limited and should be considered when planning studies in different settings.

## Conclusions

The results showed that the survivors of gynecological cancer from the LETSGO pilot felt safe and were generally satisfied with the nurse-led consultations reinforced with eHealth technology. The participants felt the nurse-led consultations and symptom registration in the app were reassuring, and they expressed an increased awareness of symptom monitoring and the benefits of physical activity. However, the objective of nurse-led care and active eHealth technology provided to empower patients and encourage self-management in the survivorship trajectory could have been more strongly emphasized. The LETSGO app was used regularly during the trial, but unexpected software issues and perceived app limitations led to reduced use among some. Participants who felt well also indicated that a survivorship app was unnecessary. Thus, a reasonable next step would be to conduct additional research using prospective intervention studies with eHealth technology and alternating care between nurses and physicians. The current study demonstrates the importance of thorough testing of new electronic applications to prevent technical challenges and inferior treatment when the app loses function.

### Practical Implications

Follow-up care with eHealth technology and nurse-led consultations is a promising approach for survivors of gynecological cancer and should be tested in a larger study that compares such care with the traditional follow-up program. The present study includes patient perspectives on the implementation of nurse-led consultations and eHealth technology in a follow-up setting. The opinions, suggestions, and needs of the participants in the current study should be considered when planning larger intervention studies on personalized care to help prevent patient dissatisfaction and reduce dropout rates.

## Supplementary Information

**Additional file 1.**

## Data Availability

The data that support the study findings are available from the corresponding author upon reasonable request.

## Abbreviations

eHealth technology: Electronic technology used in the health care system
FIGO: International Federation of Gynecology and Obstetrics
LETSGO: Lifestyle and Empowerment Techniques in Survivorship of Gynecologic Oncology
