# Supplementary material for: Nurse-led consultations reinforced with eHealth technology: a qualitative study of the experiences of patients with gynecological cancer
Source: BMC Nurs. 2022 Nov 25;21:326. doi: 10.1186/s12912-022-01104-9 (PMC9701034; doi:10.1186/s12912-022-01104-9)
Supplement: Supplementary file 1 — Additional file 1. [file 12912_2022_1104_MOESM1_ESM.pdf]

Appendix 1: Interview guide for the pilot study participants

Please tell us why you joined this study and your experiences in the participation.

- Best parts?
- Worst parts?

What were your experiences and thoughts on the nurse-led consultations?

What were your experiences and thoughts on the physician-led consultations?

Did you feel well taken care of? Please elaborate.

- What is your opinion on the length of the consultations?
- Was the goal setting meaningful?

To what degree did you perceive the provided information as relevant?

Was there any information you found lacking?

You were encouraged to perform physical activity – what are your thoughts on this matter?

What were the physical activity goals you set for yourself?

What would be the most motivating factors for engaging in physical activities in your opinion?

Did you become more physically active in the study period?

What are your thoughts on the mobile app?

- What worked well or less well?
- Did you use the app frequently?
- What parts of the app did you use most frequently?

What are your perception on receiving the symptom scoring questionnaire once a month?

If you received an alarm from the symptom scoring, how did you react and what did you do?

What are your experiences with the activity tracker?
